# Epigenetic regulation of triple negative breast cancer (TNBC) by TGF-β signaling

**DOI:** 10.1038/s41598-021-94514-9

**Published:** 2021-07-29

**Authors:** Radhakrishnan Vishnubalaji, Nehad M. Alajez

**Affiliations:** 1 Translational Cancer and Immunity Center (TCIC), Cancer Research Center, Qatar Biomedical Research Institute (QBRI), Hamad Bin Khalifa University (HBKU), Qatar Foundation (QF), P.O. Box 34110, Doha, 00000 Qatar; 2 College of Health and Life Sciences, Hamad Bin Khalifa University (HBKU), Qatar Foundation (QF), Doha, Qatar

**Keywords:** Breast cancer, Long non-coding RNAs, miRNAs

## Abstract

TGFβ signaling plays crucial role during development and cancer, however the role for TGFβ signaling in regulating the noncoding part of the human genome in triple negative breast cancer (TNBC) is still being unraveled. Herein, we provide the transcriptional landscape of TNBC in response to TGFβ activation and subsequent inhibition employing SB431542, selective TGFβ1 Receptor ALK5 Inhibitor. Our data revealed 72 commonly upregulated [fold change (FC) ≥ 2.0], including PLAU, TPM1, TAGLN, COL1A1, TGFBI, and SNAI1, and 53 downregulated (FC ≤ 2.0) protein coding genes in BT-549 and MDA-MB-231 models in response to TGFβ1 activation. Alignment to the geocode (V33) identified 41 upregulated (FC ≥ 2.0) and 22 downregulated (FC ≤ 2.0) long non-coding RNA (lncRNA) in response to TGFβ1 activation, which were inhibited by concurrent treatment with SB431542. To place our data from the in vitro models into their clinical context, we identified AC015909.1, AC013451.1, CYP1B1-AS1, AC004862.1, LINC01824, AL138828.1, B4GALT1-AS1, AL353751.1, AC090826.3, AC104695.4, ADORA2A-AS1, PTPRG-AS1, LINC01943, AC026954.3, TPM1-AS, ZFPM2-AS1, AC007362.1, AC112721.2, MALAT1, AL513314.2, AC112721.1, AC010343.3, LINC01711, and MAP3K2-DT lncRNA expression to positively correlate with TGFβ1 expression in a cohort of 360 TNBC patients. To provide mechanistic insight into lncRNA regulation by TGFβ signaling, SMAD2/3 ChIp-Seq data from BT-549 TNBC model retrieved from Gene Expression Omnibus (GEO) revealed direct binding of SMAD2/SMAD3 to the promoter of AC112721.1, AC112721.2, MALAT1, HHIP-AS1, LINC00472, and SLC7A11, suggesting their direct regulation by TGFβ1/SMAD2/SMAD3 pathway. Interestingly, AC112721.1, AC112721.2 exhibited higher expression in TNBC compared to normal breast tissue suggesting a possible role for those lncRNA in TNBC biology. Our miRNA analysis in the BT-549 model in response to exogenous TGFB1 revealed several affected miRNAs (2.0 ≤ FC ≤ 2.0), whose expression pattern was reversed in the presence of SB431542, suggesting those miRNA as plausible targets for TGFβ regulation. In particular, we observed hsa-miR-1275 to be downregulated in response to TGFB1 which was highly predicted to regulate PCDH1, FIBCD1, FXYD7, GDNF, STC1, EDN1, ZSWIM4, FGF1, PPP1R9B, NUAK1, PALM2AKAP2, IGFL3, and SPOCK1 whose expression were upregulated in response to TGFβ1 stimulus. On the other hand, hsa-miR-181b-5p was among the top upregulated miRNAs in response to TGFB1, which is also predicted to regulate CDKN1B, TNFRSF11B, SIM1, and ARSJ in the BT-549 model. Taken together, our data is the first to provide such in depth analysis of lncRNA and miRNA epigenetic changes in response to TGFβ signaling in TNBC.

## Introduction

Tumorigenesis is a complex process driven by numerous genetic alterations, altered signaling pathways, and activation of transcription factors over an extended period of time ultimately transforming normal into a cancerous cell. Predominantly, multidimensional signal transduction cascades regulate normal cellular homeostasis, including the transforming growth factor-beta (TGF-β) pathway. TGFβ appears to play dual role as a tumor suppressor in early stage of cancer progression and tumor promotor by enhancing tumorigenesis that undergo epithelial–mesenchymal transition (EMT) and eventually leads to chemo-resistant metastatic cancer at the advanced stage^1–3^. TGF-β is the prototypic super family members of over 30 secreted large members comprising an extended structurally associated group of cytokines/regulatory polypeptides. Including, TGF-βs, bone morphogenetic proteins (BMPs), growth and differentiation factors (GDFs), activins (ACTs), inhibins (INHs), glial-derived neurotrophic factors (GDNFs) and nodal growth differentiation factor (Nodal). TGF-β is one among the major regulators of cellular homeostasis in multicellular organisms, which binds to two different membranous serine/threonine kinase receptors TGF-β type II and TGF-β type I (activin-like kinase 5, ALK5). Upon ligand binding, TGF-β -type II receptor phosphorylate TGF-β-type I receptor, subsequently translocate SMAD complex into the nucleus and ultimately regulates various target genes including FOXH1, MIXER, RUNX-proteins and E2F^4–6^.

TGF signaling has well documented role in tumorigenesis. TGF-β and BMP2 signaling plays significant roles throughout breast cancer (BC) development and promote metastasis by triggering bone metastasis genes IL11 and CTGF in vivo^7,8^. Furthermore, TGF-β was found to prime tumor cells for pulmonary metastasis through initiation of angiopoietin-like 4 (ANGPTL4) via SMAD pathway in BC microenvironment^9^.

Despite the conspicuous role of TGF-β in cellular development and pathogenesis, the role of TGFβ signaling in regulating non-coding RNAs is still being unraveled. Data in the literature suggested an interplay between miRNA and TGFβ signaling including miR-34 and miR-203/SNAIL1, miR-200 and miR-205/ZEB, miR-1 and miR-200/SLUG and the autocrine TGF-β/miR-200 negative feedback loops identified at the core of TGF-β induced EMT^10^.

Similarly, long non-coding RNAs (lncRNAs) are also involved in TGF-β mediated EMT. LncRNAs can exert their function as chromatin modifiers and though regulation of various mRNAs by competitively binding to microRNAs (miRNAs). Limited studies have shown the functional effects of LncRNAs in the context of TGF-β signaling in various cancers. Amongst, few studies have identified lncRNAs involved in TGF-β induced EMT processes, such as lncRNA-ATB, AC026904.1, UCA1, HIT, ROR, HOXA-AS2, CCAT2, which are highly expressed, whereas NKILA, ANCR, lnc-Spry1 and MEG3 are oftentimes downregulated in BC^11^.

Recently, we have shown the association of novel LINC01614 with TGF-β and FAK signaling in HR^+^/HER2^+^ BC molecular subtype^12^. However, the role of TGF-β signaling in regulating lncRNA expression in triple negative breast cancer (TNBC) remains elusive. Herein, we provide complete picture of the cross regulation between miRNA, lncRNA, and mRNA in the context of TGFβ signaling employing transcriptome and bioinformatics analyses. We also provide direct correlation between the expression of several lncRNAs and TGFβ signaling in TNBC patients’ specimens.

## Results

### Comparative analysis of the protein coding transcriptome in response to TGFβ signaling in TNBC

**Figure 1:**
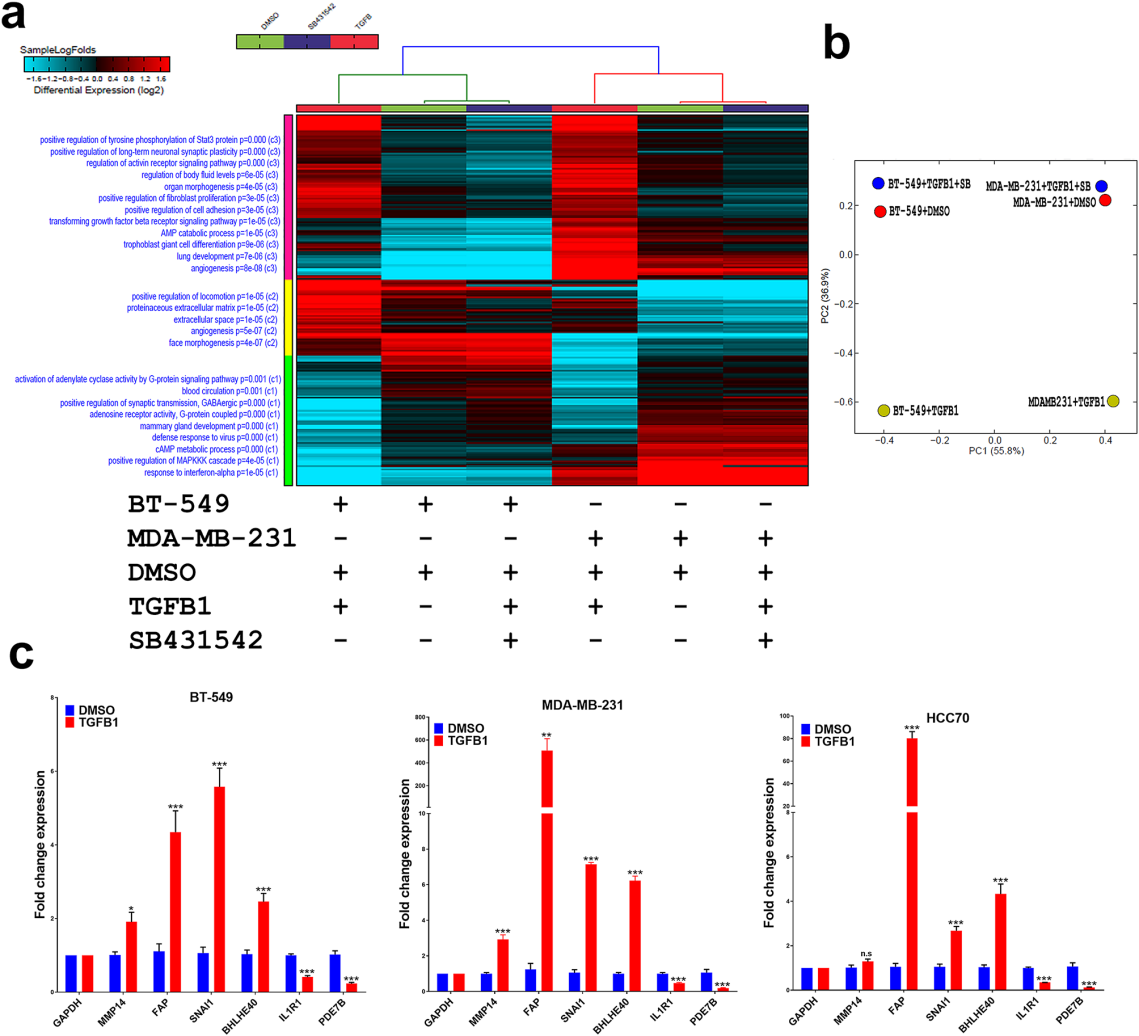
(**a**) Heatmap depicting changes in protein coding gene expression in BT-549 and MDA-MB-231 under different experimental conditions. Enriched gene ontology (GO) associations/pathways are indicated on the y axis. (**b**) Principal component analysis (PCA) for the mRNA transcriptome of BT-549 and MDA-MB-231 under different experimental conditions. (**c**) The expression of selected number of differentially expressed genes was validated using qRT-PCR in BT-549, MDA-MB-231, and HCC70 TNBC models. Data are presented as mean ± SEM (n = 6). *P < 0.05; **P < 0.005; ***P < 0.0005, *n.s.* not significant.

To characterize TGF-β-induced transcriptional alterations in TNBC, BT-549 and MDA-MB-231 models were exposed to rhTGF-β (10 ng/ml) as single agent or in combination with SB431542 (10 μM/ml), a well-known TGF-β inhibitor. Heatmap depicting differentially expressed protein coding genes under the aforementioned experimental conditions is presented in Fig. 1a where each column represents one experimental condition, and each row represents an mRNA. Expression level of each gene (log2) in a single treatment condition is depicted according to the color scale. Enriched functional annotations and pathways are illustrated on the left side of the heatmap. When comparing rhTGF-β and rhTGF-β + SB431542 to DMSO control, we observed three major common clusters in both BT-549 and MDA-MB-231. In Fig. 1a, cluster C1 in hierarchical clustering illustrate G-protein signaling pathway, blood circulation, positive regulation of synaptic transmission GABAergic, mammary gland development and defense response to virus functions were activated by rhTGF-β in BT-549 and MDA-MB-231, and on the contrary, these functional transcripts were reversed by SB431542 treatment to a level comparable to DMSO control. In addition, SB431542 reversed the effect of rhTGF-β which induced pathways of proteinaceous extracellular matrix, extracellular space and angiogenesis in cluster C2. Similar revocable effects by SB431542 were observed in regulation of tyrosine phosphorylation of stat3 protein, regulation of activin receptor signaling pathway, regulation of body fluid levels, organ morphogenesis, positive regulation of fibroblast proliferation and cell adhesion and TGF-β receptor signaling pathways (Fig. 1a). The gene expression principal component analysis (PCA) plot delivers insights into the relationship between samples; mainly we could observe the reversed effects of SB431542 through adjacent falling into DMSO condition, based on PC1, 56% and PC2, 36% of the variation attribution (Fig. 1b). The expression of selected number of differentially expressed genes (MMP14, FAP, SNAI1, BHLHE40, TGFBI, IL1R1 and PDE7B) was validated using qRT-PCR in BT-549, MDA-MB-231, and HCC70 TNBC models (Fig. 1c).

**Figure 2:**
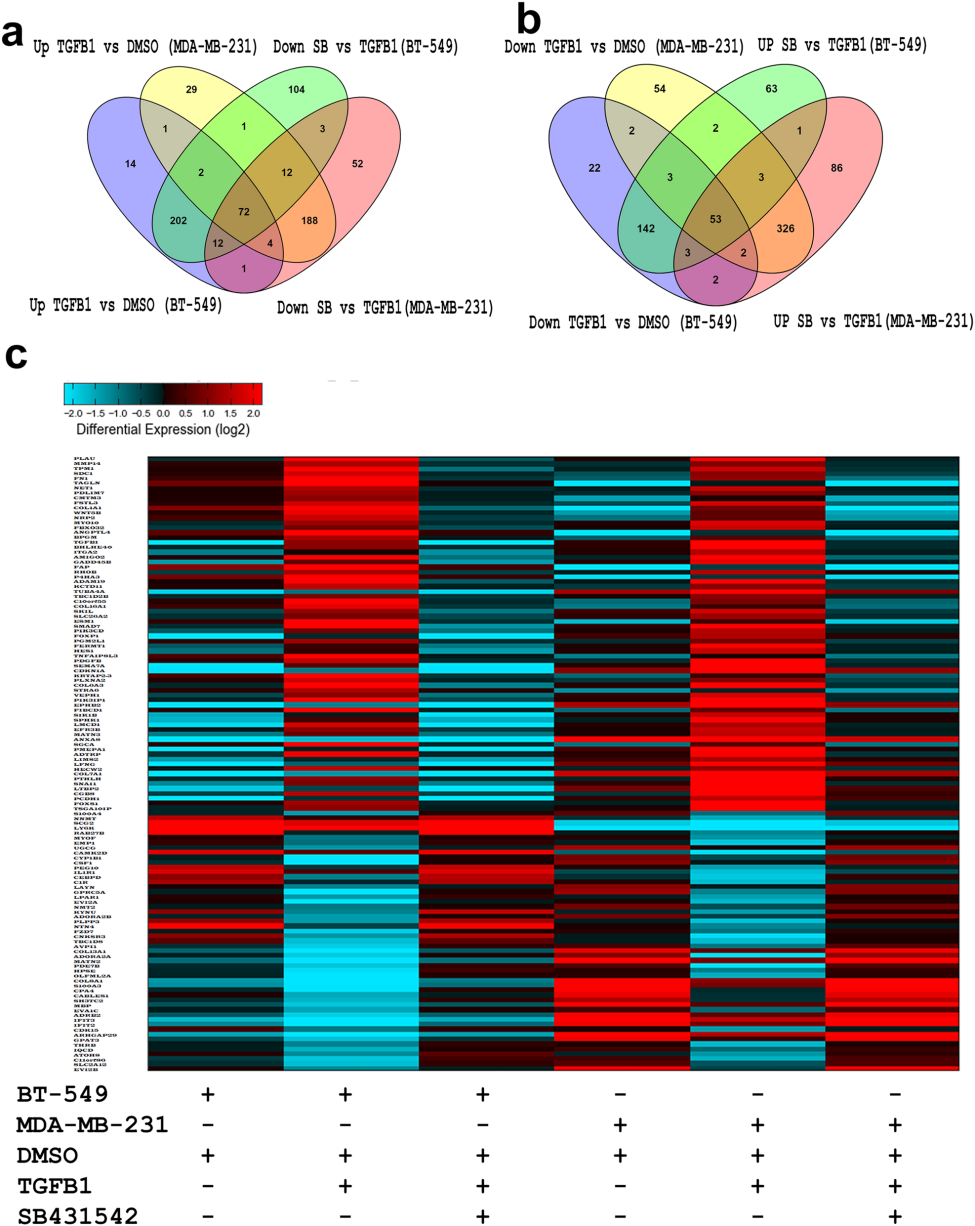
Venn diagraph depicting the overlap in upregulated (**a**) or downregulated (**b**) genes in BT-549 and MDA-MB-231 under the indicated experimental conditions. Upregulated genes are defined as the genes upregulated (FC ≥ 2.0) by TGFB and those were downregulated (FC ≤ 2.0) by SBSB431542. Downregulated genes were defined as the genes downregulated by TGFB1 (FC ≤ 2.0) and those were induced by SB431542 (FC ≥ 2.0). (**c**) Heatmap depicting commonly altered (up and down) protein coding genes under different experimental conditions.

In order to identify commonly regulated genes by TGFβ signaling, we compared the mRNA profile of BT-549 and MDA-MB-231 cells under different experimental conditions. Venn diagram depicting the overlap in upregulated (FC ≥ 2.0) and downregulated (FC ≤ 2.0) genes in both TNBC models and identified commonly 72 upregulated (FC ≥ 2.0) and 53 downregulated (FC ≤ 2.0) genes, which were reversed by SB431542 treatment (Fig. 2a,b), implying their regulation by TGFβ singling. Furthermore, heatmap depicting commonly deregulated protein-coding genes under different experimental conditions, respectively (Fig. 2c, supplementary table 1). Not surprisingly, several of the identified gene targets are associated with TGF-β/SMAD2/SMAD3 pathway, such as PLAU, TPM1, TAGLN, COL1A1, TGFBI, and SNAI1.

### Effects of TGFβ signaling on lncRNA transcriptome in TNBC

**Figure 3:**
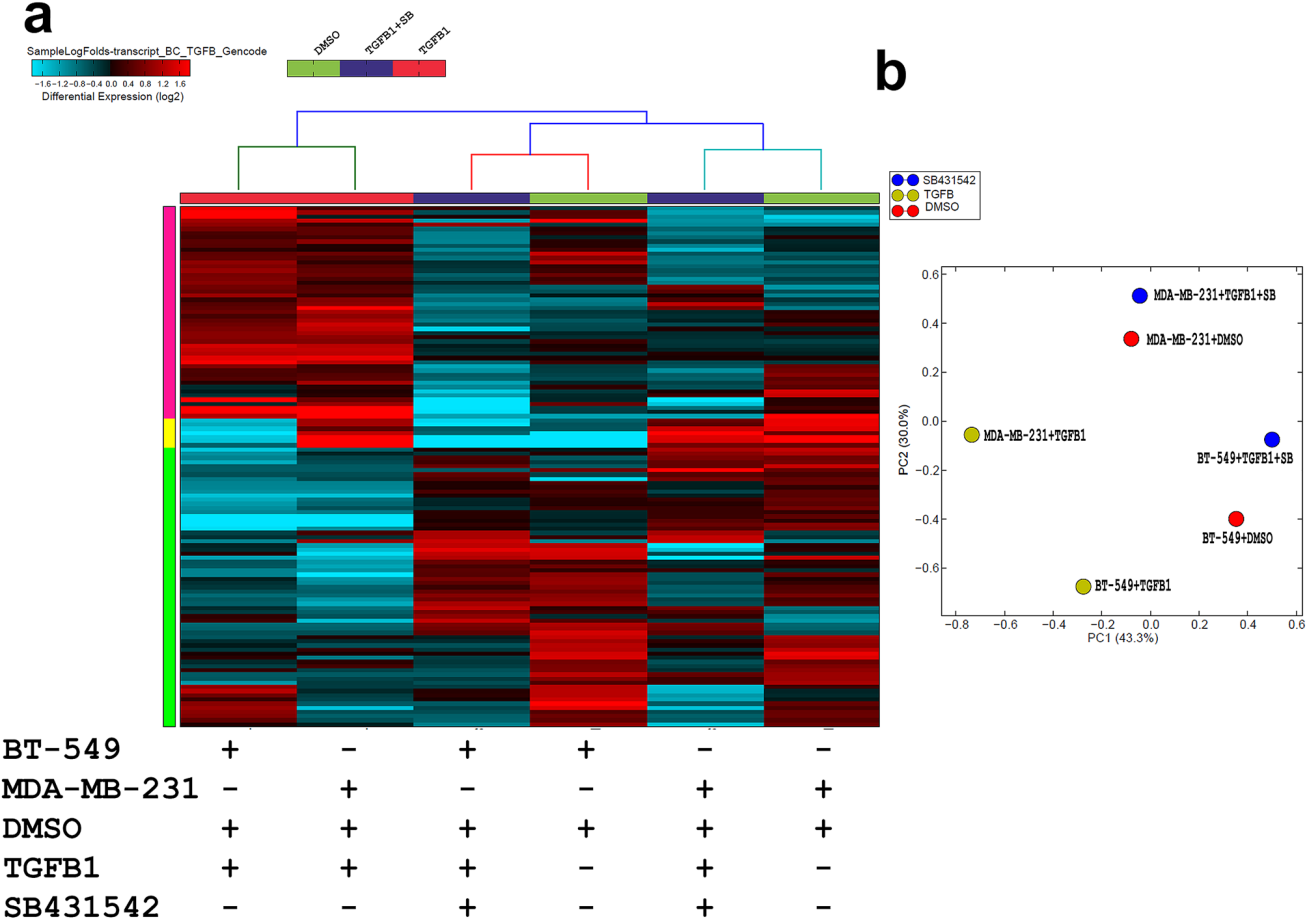
(**a**) Heatmap depicting changes in lncRNA expression under different experimental conditions. (**b**) PCA analysis.

**Figure 4:**
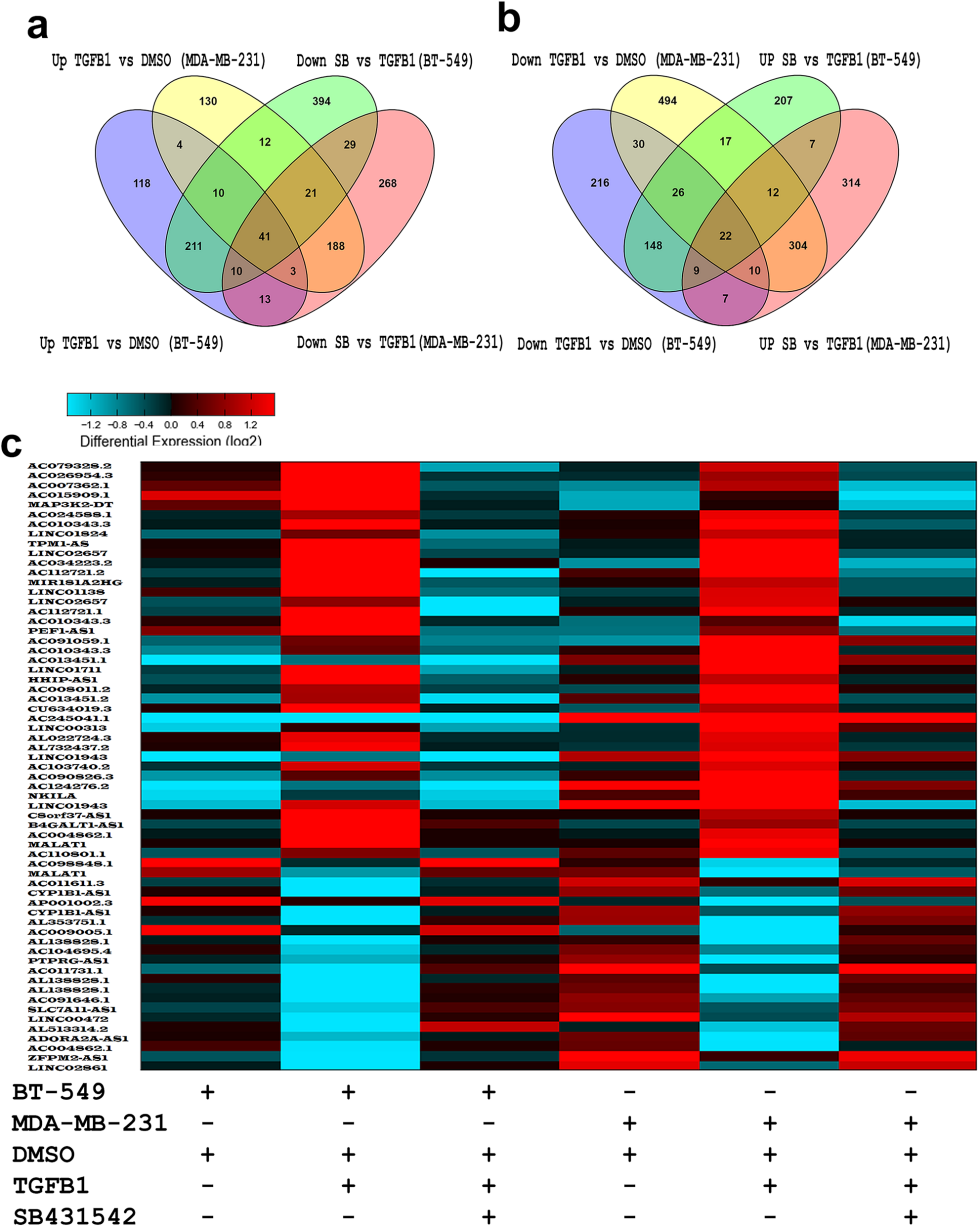
Venn diagraph depicting the overlap in upregulated (**a**) or downregulated (**b**) lncRNAs in BT-549 and MDA-MB-231 under the indicated experimental conditions. Upregulated genes are defined as the genes upregulated (FC ≥ 2.0) by TGFB1 and those were downregulated (FC ≤ 2.0) by SBSB431542. Downregulated genes were defined as the genes downregulated (FC ≤ 2.0) by TGFB1 and those were induced (FC ≥ 2.0) by SB431542. (**c**) Heatmap depicting commonly altered (up and down) lncRNAs under different experimental conditions.

We subsequently sought to characterize alterations in the lncRNA transcriptome in response to TGFβ signaling. Figure 3a provides hierarchical clustering of differentially expressed lncRNAs in BT-549 and MDA-MB-231 models where each column represents specific treatment condition, and each row represents an lncRNA expression (log2) where the expression is depicted according to the color scale (supplementary table 2). The lncRNAs expression PCA confirmed the relationship between different treatment showing close proximity between rhTGF-β + SB431542 and DMSO, which are distantly displaced from the rhTGF-β group on PC1, 43% and PC2, 30% of the variation attribution (Fig. 3b). Subsequently, we sought to identify commonly dysregulated lncRNAs in response to TGFβ1 stimulation, which also were reversed by concurrent treatment with SB431542 in both TNBC models. Venn diagram depicting the overlap in up and downregulated lncRNAs in both TNBC models identified 41 commonly upregulated (FC ≥ 2.0) and 22 downregulated (FC ≤ 2.0) lncRNAs (Fig. 4a,b). Illustration of the differentially expressed lncRNAs in BT-549 and MDA-MB-231 TNBC models is shown as heatmap (Fig. 4c, supplementary table 3).

### Correlation between lncRNA expression and TGFβ signaling in a large cohort of TNBC patients

**Figure 5:**
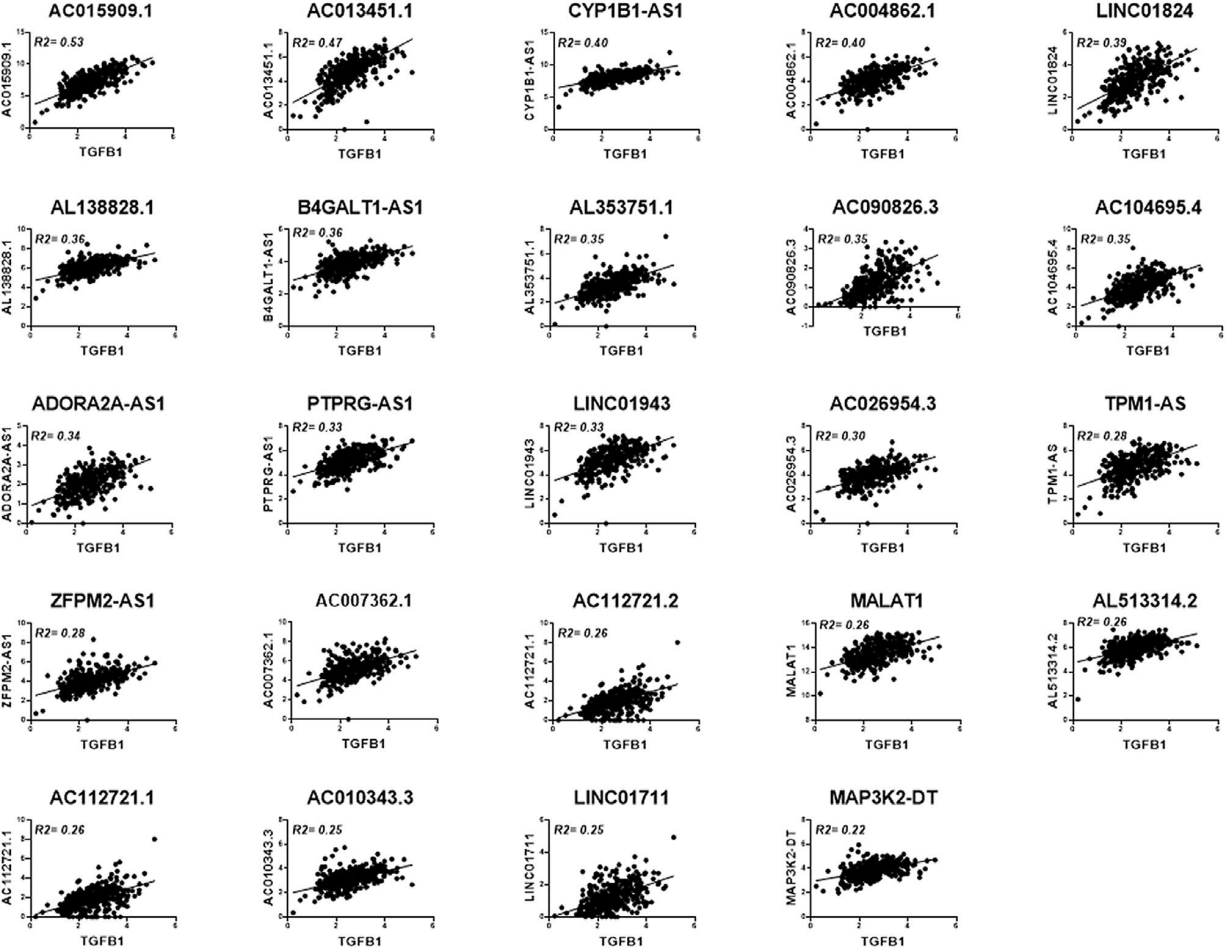
Dot plot depicting the correlation between the expression of TGFB1 and the indicated lncRNAs in a cohort of 360 TNBC patients.

In order to place our findings from the in vitro models into their clinical context, we investigated the correlation between TGFβ signaling and the expression of the differentially expressed lncRNAs employing a cohort of 360 TNBC patients previously described^13^. Our analysis revealed positive correlations (R^2^ ≥ 0.2) between TGFβ1 expression and the expression of AC015909.1, AC013451.1, CYP1B1-AS1, AC004862.1, LINC01824, AL138828.1, B4GALT1-AS1, AL353751.1, AC090826.3, AC104695.4, ADORA2A-AS1, PTPRG-AS1, LINC01943, AC026954.3, TPM1-AS, ZFPM2-AS1, AC007362.1, AC112721.2, MALAT1, AL513314.2, AC112721.1, AC010343.3, LINC01711 and MAP3K2-DT. Therefore, our analysis underlining a plausible correlation between TGFβ signaling the aforementioned lncRNAs in TNBC (Fig. 5).

### ChIP-seq analysis provides support of direct binding of SMAD2/SMAD3 to the promoter region of several TGF-β-dependent lncRNAs

**Figure 6:**
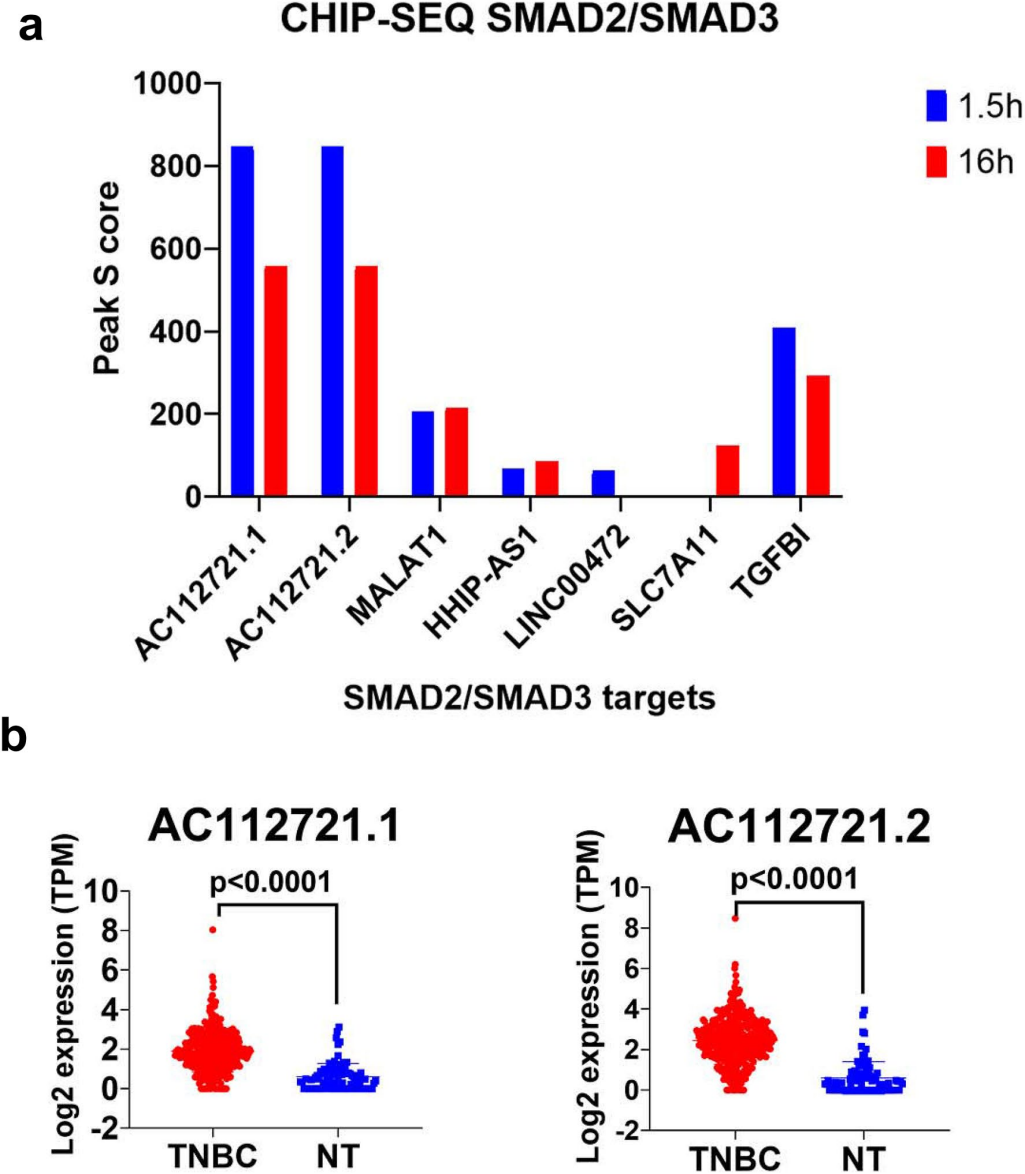
(**a**) ChIp-Seq analysis demonstrating direct binding of SMAD2/SMAD3 to the promoter region of the indicated lncRNAs. TGFBI was used as positive control. (**b**) Expression of AC112721.1 and AC112721.2 in a cohort of 360 TNB and 88 normal breast tissue.

In order to provide direct evidence showing regulation of the identified lncRNAs from current study by SMAD2/SMAD3 singling, we analyzed chromatin immunoprecipitation (using SMAD2/SMAD3 ab) and sequencing (ChIP-seq) data from the BT-549 cell model previously described^14^. Data presented in Fig. 6a revealed direct binding of SMAD2/SMAD3 to the promoter region of the AC112721.1, AC112721.2, MALAT1, HHIP-AS1, LINC00472, SLC7A11, along with TGFβI as positive control, suggesting their direct regulation by TGFβ1/SMAD2/SMAD3 pathway in BT-549. AC112721.1, AC112721.2 exhibited highest peak score and higher  expression levels of both lncRNAs was observed in TNBC patients (n = 360) compared to controls (n = 88, Fig. 6b).

### Integrated analysis of miRNA and mRNA regulatory networks in response to TGFβ signaling in TNBC

**Figure 7:**
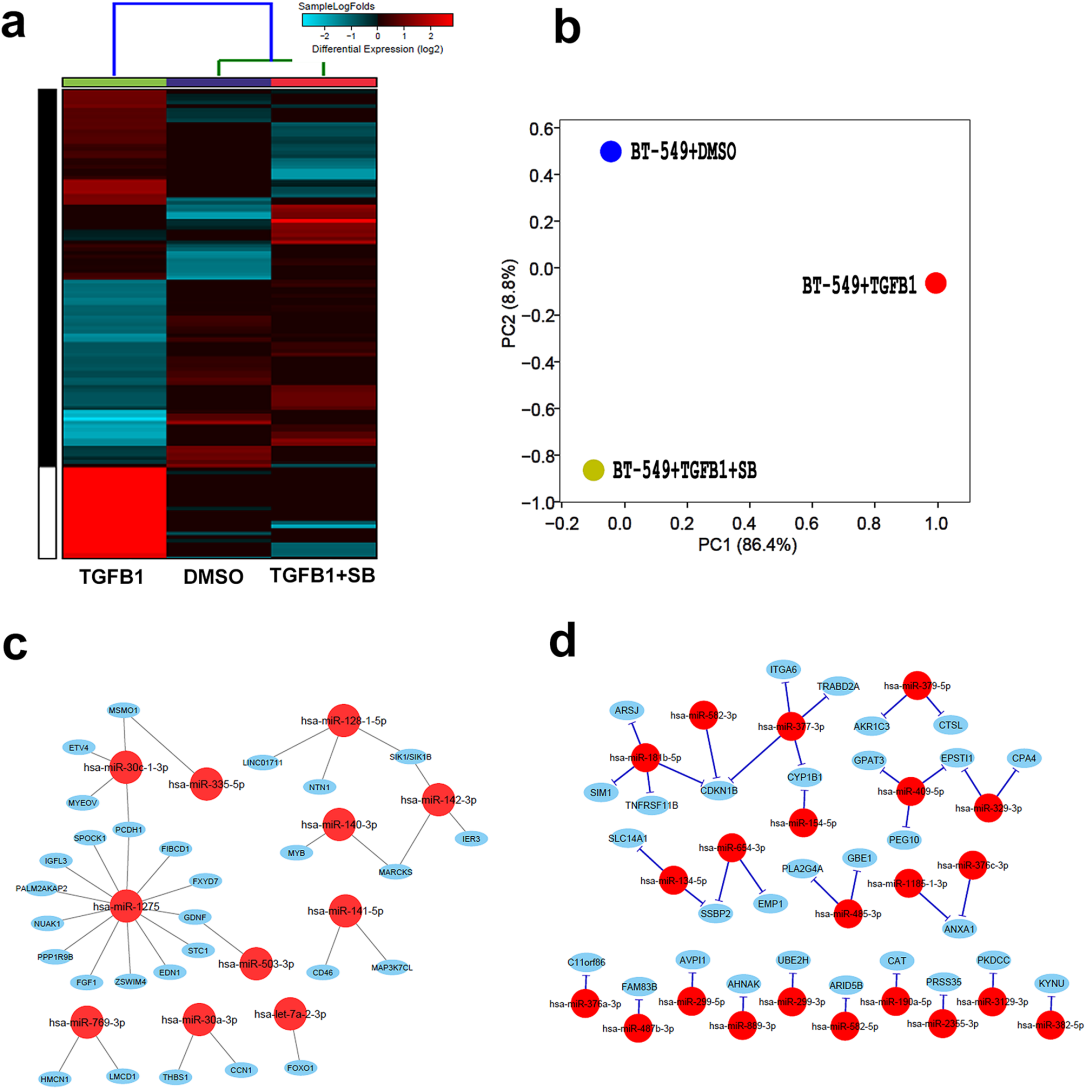
(**a**) Heatmap depicting changes in miRNA expression under different experimental conditions. (**b**) PCA analysis. (**c**) Highly predicted or validated mRNA targets of up (left) and down (right) based on Targetscan and IPA perdition that are affected in BT-549 under different experimental conditions.

To identify potential miRNA–mRNA regulatory networks triggered by TGFβ in TNBC, we performed miRNA sequencing in BT-549 model in response to rhTGF-β (10 ng/ml) as single agent or in combination with SB431542 (10uM/ml). Our data revealed 37 upregulated (FC ≥ 2.0) and 19 downregulated (FC ≤ 2.0) miRNAs whose expression could be reversed by SB431542 treatment (Fig. 7a,b). Data from miRNA and mRNA expression were subsequently integrated using the IPA microRNA target filter, which utilizes complex target prediction and validation databases, to provide deeper insight into the miRNA–mRNA regulatory networks. Our analysis revealed the pairing relationship of downregulated hsa-miR-1275, hsa-miR-30c-1-3p, hsa-miR-335-5p, hsa-miR-128-1-5p, hsa-miR-142-3p, hsa-miR-140-3p, hsa-miR-141-5p, hsa-miR-503-3p, hsa-miR-769-3p, hsa-miR-30a-3p and hsa-let-7a-2-3p and potential binding to several upregulated mRNAs in rhTGF-β treated BT-549 cells (Fig. 7c, supplementary table 4). For instance, downregulated hsa-miR-1275 exhibited pairing with 30 upregulated mRNAs associated with apelin cardiac fibroblast signaling pathway, actin cytoskeleton signaling, dendritic cell maturation, adipogenesis pathway, HOTAIR regulatory, cardiac hypertrophy signaling (enhanced), breast cancer regulation by Stathmin1, axonal guidance signaling, cAMP-mediated signaling, cardiac β-adrenergic signaling and protein kinase A Signaling. Likewise, hsa-141-5p also has binding relationship with 11 mRNAs associated with human embryonic stem cell pluripotency, TGF-β signaling, complement system and some of pathways which are similar to has-miR-1275 (supplementary table 4). Likewise, IPA analysis of high-confidence interaction revealed the pairing relationship of upregulated hsa-miR-181b-5p, hsa-miR-582-5p, hsa-miR-377-3p, hsa-miR-154-5p, hsa-miR-379-5p, hsa-miR-409-5p, hsa-miR-329-3p, hsa-miR-134-5p, hsa-miR-654-3p, hsa-miR-485-3p, hsa-miR-1185-1-3p, hsa-miR-376a-3p, hsa-miR-376c-3p, hsa-miR-487b-3p, hsa-miR-299-3p, hsa-miR-889-3p, hsa-miR-299-3p, hsa-miR-582-5p, hsa-miR-190a-5p, hsa-miR-2355-3p, hsa-miR-3129-3p and hsa-miR-382-5p and numerous downregulated mRNA targets (Fig. 7d, supplementary table 4). Most of the mRNAs are associated with integrin signaling, leukocyte extravasation signaling, NF-κB activation by viruses, role of tissue factor in cancer, breast cancer regulation by Stathmin1, eicosanoid signaling and androgen biosynthesis signaling pathway (supplementary table 4). miRNA–mRNA network analyses in the MDA-MB-231 model revealed common networks (hsa-miR-212-3p, hsa-miR-181a-2-3p, and hsa-miR-582-3p) however each cell model also displayed their networks (Supplementary table 5).

## Discussion

TNBC represent an aggressive subtype of BC which lack expression of common targets such as estrogen, progesterone and human epidermal growth factor receptors for targeted therapies^15^. In complex disease, tumor heterogeneity and reciprocal connection between tumor cells and adjacent cell populations like stromal cells, cancer-associated fibroblasts (CAFs) and extracellular matrix are major factors that regulate tumorigenesis and treatment response. Particularly, CAF in axillary lymph nodes drives metastases in BC through corresponding mechanisms, especially EMT that involves TGF-β pathway^16^, although the role of TGF-β in TNBC is not fully understood. The relationship between TGF-β pathways, especially downregulation of TGF-β receptors, indicates the aggressive BC phenotype and stipulates contradictory correlation with patient’s outcomes^17^. A previous study showed that intratumoral high expression of TGF-β1 was observed in TNBC (52.5%) compared with non-TNBC (27.5%), implying plausible role for TGFβ signaling in TNBC biology. TGF-β1 was found to enhance the tumorigenic property of MDA-MB-231 cells through activating SMAD2 and P38 signaling pathways. Overall, these studies indicate the association of TGF-β1 with poor disease-free survival, aggressiveness, and metastasis status of BC^18,19^.

In current study we provide the transcriptional portrait of mRNA. lncRNA, and miRNA in the context of TGFB1 activation and reversal by SB431542 in two TNBC models. Our data identified 71 commonly upregulated and 53 downregulated mRNAs in response to TGFB1 activation, which were also reversed by concurrent treatment with TGFB1 and SB431542, implying their regulation by TGFB pathway. Several of the identified genes are known TGFβ/SMAD2/SMAD3 targets, such as PLAU, TPM1, TAGLN, COL1A1, TGF-βI, and SNAI1 thus validating our experimental system^1,20^. A number of functional categories were enriched in response to TGF-β treatment in both TNBC models, including proteinaceous extracellular matrix, extracellular space and angiogenesis, tyrosine phosphorylation of stat3 protein, activin and TGFβ receptor signaling pathway, regulation of body fluid levels, organ morphogenesis, positive regulation of fibroblast proliferation and cell adhesion pathways. Activin belongs to TGF-β super family and Activin-A correlates with poor survival rate in advanced BC. Furthermore, Activin-A enhance anchorage-independent growth, angiogenesis, invasion, stemness and EMT of BC through SMAD signaling pathway^21^. The reversible effects of SB431542 confirmed the association of these functional categories to TGF-β signaling.

While the functional role of TGF-β in BC progression has been investigated rigorously through gene regulation and phenotypic changes, the role of TGF-β pathway in regulating the noncoding part of the human genome is still being unraveled. our data revealed aberrant expression of TGF-β-regulated lncRNAs and miRNAs. Herein we identified 41 upregulated and 22 downregulated lncRNAs in response to TGFβ signaling. Our in vitro findings were subsequently validated in a cohort of 360 publicly-available TNBC patient dataset. Twenty-four lncRNAs strongly correlated with TGFβ1 expression in TNBC tissue. AC015909.1, AC013451.1, CYP1B1-AS1, AC004862.1, LINC01824, AL138828.1, B4GALT1-AS1, AL353751.1, AC090826.3, AC104695.4, ADORA2A-AS1, PTPRG-AS1, LINC01943, AC026954.3, TPM1-AS, ZFPM2-AS1, AC007362.1, AC112721.2, MALAT1, AL513314.2, AC112721.1, AC010343.3, LINC01711, and MAP3K2-DT showed highest correlation with TGFβ signaling. To provide direct evidence of the regulation of those lncRNAs by TGFβ signaling, we explored GEO ChIP-seq dataset and identified direct binding of AC112721.1, AC112721.2, MALAT1, HHIP-AS1, LINC00472 and SLC7A11 promoters by SMAD2/SMAD3, thus providing evidence of direct regulation of those lncRNAs by TGFβ through SMAD2/SMAD3 binding.

In addition to lncRNA, we also characterized the miRNA transcriptome regulated by TGFβ signaling in TNBC. hsa-miR-382-5p, hsa-miR‐376c-3p, and hsa-miR‐379-5p were the top three miRNAs that highly upregulated by rhTGF-β and those were reversed by simultaneous treatment with rhTGF-β and SB431542. Interestingly, hsa-miR-382-5p was previously reported to exert oncogenic function augmenting the tumorigenic and metastatic potential of BC through diminish the tumor suppressive role of RERG (Ras-related and estrogen-regulated growth inhibitor), a Ras GTPase superfamily member on the oncogenic Ras/ERK pathway^22^. We observed hsa-miR-181a-5p to be upregulated in BT-549 and MDA-MB-231 cells in response to TGFβ stimulation. hsa-miR-181a-5p was previously shown to hinder cell cycle inhibitor CDKN1B and other genes such as ARHGEF3, SLC2A3, SSBP2, TNFRSF11B, PHLDA1, SIM1, ARSJ, EVI2A and PLPP3. Oncogenic role of hsa-miR‑181was also reported in BC through the targeting SPRY4^23^. Furthermore, knockdown of SOX2 decreased the level of cell cycle protein CCND1, CDK4 and CDK6, and reduced the expression of hsa-miR-181a-5p and hsa-miR-30e-5p in MDA-MB-231^24^. Previous study has shown four miRNAs including hsa-miR‐148b, hsa-miR‐376c, hsa-miR‐409‐3p and hsa-miR‐801, which were upregulated in the plasma of BC patients^25^. In current study we found hsa-miR‐376c and hsa-miR‐409‐3p to be highly induced by rhTGF-β treatment in TNBC.

In addition to the upregulated miRNAs, our data also identified several downregulated miRNAs in response to TGFβ activation., including hsa-miR-140-3p, hsa-miR-128-1-5p and hsa-miR-141-5p. Loss of those miRNAs upregulates INHBA (inhibin; activin A) and VDR (vitamin D receptor), which are associated with TGF-β/SMAD signaling pathways.

In current study, we observed the downregulation of hsa-miR-128-1-5p in response to TGF-β signaling. Previous study reported loss of hsa-miR-128 as one mechanism through which breast tumor–initiating cells (BT-IC) can resist doxorubicin, through regulation of Bmi-1 and ABCC5 multidrug resistance protein^26^. hsa-miR-128a was also found to negatively target TGF-βR1 in letrozole-resistant BC cells^27^. Likewise, hsa-miR-34 family inhibits apoptotic functions through regulation of a wide-ranging of pro-apoptotic and oncogenes controllers including SMAD4, NOTCH1 and TP53 in metastatic BC^28^. Our revealed downregulation of hsa-miR-34c-3p in response to TGFβ stimulation and predicted KRTAP2-3/KRTAP2-4 and MSMO1 (cholesterol biosynthesis) as potential target. It remains however to be investigated if TGFβ regulates those miRNAs directly or indirectly. While data presented in current study was focused on TNBC, the effects of TGFβ signaling on miRNA and lncRNA expression in breast cancer with other molecule subtypes remains to be investigated.

In closing, our data provide the first insight into epigenetic regulation of lncRNA and miRNA by TGFβ signaling in TNBC and suggest their potential utilization as disease biomarkers and therapeutic targets.

## Materials and methodology

### Cell culture, recombinant TGF-β treatment, and small molecule inhibition

Human BT-549 and MDA-MB-231 TNBC models were cultured in Dulbecco’s modified Eagle’s medium supplemented with d-glucose 4500 mg/l, 4 mM l-glutamine and 110 mg/l sodium pyruvate, 10% fetal bovine serum and 1 × penicillin–streptomycin (Pen-Strep) (all purchased from vGibco-Invitrogen, Waltham, MA, USA). Both TNBC models were cultured in 6 well plates (duplicate), 0.2 × 10^6^ cells/well and treated with rhTGF-β1 (10 ng/ml, Peprotec, London, UK), TGF-β inhibitor (SB431542;10 μM, Selleckchem Inc., Houston, TX, USA), and combination of rhTGF-β1 and TGF-β inhibitor for 48 h and subsequently RNA was isolated. Experiment were carried out with DMSO control^12^.

### RNA isolation and quality assessment

RNA was isolated using the RNA/DNA/Protein Purification Plus Kit (Norgen Biotek Corp, Ontario, Canada) as per the manufacturer’s instructions. Briefly, cells pellets were resuspended in the lysis buffer followed by RNA was extracted under appropriate environment. The concentration and purity of extracted RNA were measured using NanoDrop 2000c (Thermo scientific, MA, USA) and RNA were stowed at − 80 °C. The concentration and quality of RNA was analyzed using capillary electrophoresis using Agilent RNA 6000 Nano Kit (Agilent Technologies, CA, USA) and Agilent 2100 Bioanalyzer (Agilent Technologies) as per the manufacturer’s instructions. Samples with RNA Integrity Number (RIN) > 8 were considered for library preparation^29^.

### Total RNA library preparation and RNA sequencing

Total RNA samples with a RIN higher than 8 were used as input for the library preparation by using TruSeq Stranded Total RNA Library Prep Gold kit (Cat #: 20,020,598) from Illumina following the manufacturer’s protocol. Briefly, 500 ng of total RNA was subjected to rRNA depletion and then to fragmentation. The first-strand cDNA synthesis was performed with random hexamers and SuperScript II Reverse Transcriptase (Cat#: 18064014) from ThermoFisher Scientific. The second cDNA strand synthesis was performed by substitution of dTTP with dUTP. The double-stranded cDNA is then end-repaired and adenylated. Barcoded DNA adapters were ligated to both ends of the double-stranded cDNA and then amplified. The libraries quality was checked on an Agilent 2100 Bioanalyzer system and quantified using Qubit 2.0 fluorometer (Invitrogen). The libraries were pooled, clustered on a cBot platform, and sequenced on an Illumina HiSeq 4000 at a minimum of 50 million paired end reads (2 × 75 bp) per sample^29^.

### RNA-Seq data analysis of TGFB1 and SB431542 treated TNBC models

FASTQ files were subsequently pseudo aligned to the Gencode release 33 index (mRNA and lncRNA) and reads were subsequently counted using KALLISTO 0.42.1^30^ as we described before^31,32^. Normalized TPM (Transcripts Per Million) expression values were subsequently subjected to differential analysis and hierarchical clustering using cosine for columns and cosine for rows, while gene set and pathway enrichment were performed using Gene Ontology (GO)-Elite algorithm in AltAnalyze v.2.1.3 as described before^33^. MicroRNA analysis was carried out in CLC genomics workbench 20.0 using built-in small RNA analysis workflow. miRNA count reads were normalized using the TMM (trimmed mean of M values) normalization method and log2 CPM (Counts per Million) values were subsequently subjected to differential analysis. The microRNA Target Filter in IPA was subsequently employed to identify potential miRNA–mRNA networks. This bioinformatics approach provides insights into the biological effects of microRNAs, using corresponding mRNA expression data as well as experimentally validated interactions from TarBase and miRecords, and the predicted microRNA–mRNA interactions from TargetScan. Only highly predicted and experimentally predicted networks were included in final analysis. DIANA-LncBase v3 was utilized to identify miRNA–lncRNA interactions. Cytoscape 3.8.1 was used to construct miRNA–mRNA and miRNA–lncRNA networks.

### Quantitative reverse transcription PCR (qRT-PCR)

Five-hundred ng of RNA was reverse transcribed to cDNA using High Capacity cDNA Reverse Transcription kit (Applied Biosystems, Foster City, CA, USA). Real time PCR was performed using PowerUp SYBR Green Master Mix (Applied Biosystems) on QuantStudio 7/6 Flex qPCR (Applied Biosystems) using the following primer pairs: MMP14 F: CGAGGTGCCCTATGCCTAC, MMP14 R: CTCGGCAGAGTCAAAGTGG; FAP F: TGTGCATTGTCTTACGCCCT, FAP R: CCGATCAGGTGATAAGCCGT; SNAI1 F: CCTCCCTGTCAGATGAGGAC, SNAI1 R: CCAGGCTGAGGTATTCCTTG; IL1R1 F: GAGCGGCAGGAATGTGACAA, IL1R1 R: GAGGGTGCGTCTACCTGGA, BHLHE40 F: GACGGGGAATAAAGCGGAGC, BHLHE40 R: CCGGTCACGTCTCTTTTTCTC; PDE7B F: GGTTGAGAGGTGTGGCGAAAT, PDE7B R: CCTTAGTCGTATATCTCCCAGCA, GAPDH F: GGAGCGAGATCCCTCCAAAAT, GAPDH R: GGCTGTTGTCATACTTCTCATGG. Relative levels of transcripts were determined using the 2^-^^ΔΔCT^ Method relative to GAPDH reference gene.

### miRNA sequence library preparation

MiRNA library was prepared using QIAseq miRNA Library Kit (Qiagen). Before proceeding to cDNA conversion, NGS adaptor containing unique identifiers were ligated to the each 3′ and 5′ end of miRNA separately. Subsequently, NGS RT initiator, universal sequence and reverse transcription (RT) primer contains a combined unique molecular index (UMI) were added to the reaction, RT primer binds to a region of the 3′ adapter and enables conversion of the 3′/5′ ligated miRNA to cDNA. After RT, cDNA products were undergone cleanup procedure using a QIAseq miRNA NGS (QMN) beads. Next, library amplification was performed with HotStarTaq DNA Polymerase and wet universal forward primers that assigned with unique index (IDP). Finally, final products were cleaned with QMN bead. The reaction setup, components, concentrations and technical points were followed as per manufacturer’s instructions (QIAseq miRNA Library Kit Handbook for Illumina NGS Systems).

### TNBC RNA-Seq data retrieval and analysis

The transcriptome data were retrieved from 360 TNBC and 88 normal from the Sequence Read Archive (SRA) database (https://www.ncbi.nlm.nih.gov/sra/SRP157974) using the SRA toolkit version 2.9.2 as previously described. Clinical characteristics of the patients and controls were described before^13^. Paired end RNASeq FASTQ files were subsequently pseudo aligned to the human genome and reads were counted using KALLISTO 0.42.1^30^. The R-squared goodness-of-fit measure for linear regression models was used to identify the correlation between the expression of TGFB1 and the indicated lncRNA using Graphpad prism 8.0.

### ChIP-seq analysis

Chip-Seq peak score for SMAD2/SMAD3 from the BT-549 model was retrieved from the GSE104352 study as described by Sundqvist et al.^14^. In brief, SMAD2/3 ChIP-seq was conducted on the BT-549 TNBC cell model at 1.5 h and 15 h. ChIP-seq data sets were then aligned using Bowtie (version 1.1.0), while binding regions were identified using MACS software Model based analysis of ChIP-seq (version 1.4.2). Chip-Seq peak score peaks were retrieved from GEO.

### Statistical analysis

Correlation analysis were conducted in Graphpad Prism 8.0 software and an R^2^ ≥ 0.2 was considered significant (Graphpad Software, San Diego, CA). Two-tiled t test was used to compare analysis groups.

## Supplementary Information

Supplementary Table S1.

Supplementary Table S2.

Supplementary Table S3.

Supplementary Table S4.

Supplementary Table S5.
